# TEM8 specific T cells target the tumor cells and tumor-associated vasculature in triple negative breast cancer

**DOI:** 10.1186/2051-1426-2-S3-P7

**Published:** 2014-11-06

**Authors:** Tiara Byrd, Kristen Fousek, Antonella Pignata, Amanda Wakefield, Brad St Croix, Bradley S Fletcher, Meenakshi Hegde, Nabil Ahmed

**Affiliations:** 1 Baylor College of Medicine, Houston, TX, USA; 2 National Cancer Institute - Frederick, MD, USA; 3 University of Florida, Gainesville, FL, USA

## Background

Tumor endothelium marker 8 (TEM8) was discovered by St Croix, et. al. as one of nine gene products preferentially upregulated in the tumor-associated vs. normal endothelium [1]. Interestingly, TEM8 has also been identified as a tumor restricted antigen in triple negative breast cancers (TNBC) [2,3]; a clinical entity associated with a particularly poor prognosis. Being null for HER2, estrogen and progesterone receptors, targeted therapies for TNBC are quite limited.

## Purpose

To use T cells expressing TEM8-specific chimeric antigen receptors (CAR) as a novel approach to target both TNBC cells and their tumor-associated vasculature.

## Methods/ results

We used *in silico *design to construct a novel TEM8-specific CAR molecule. The antigen recognition exodomain consisted of a single chain variable fragment based on the TEM8-specific monoclonal antibody, SB5. The signaling endodomain consisted of the costimulatory molecule CD28 and CD3-zeta chain. The encoding DNA was codon optimized, synthesized and then sequence verified. We used a retroviral transduction system to express the TEM8 CAR transgene on HEK 293T, then on primary T cells. Approximately 70% of primary human T cells expressed the TEM8 CAR, as detected by flow cytometry. The expression of TEM8 was characterized using flow cytometry and western blot on a battery of TNBC lines, TEM8 transduced (modest and high expressers) cell lines as well as TEM8 negative cell lines. TEM8 CAR T cells recognized and killed TEM8 positive target cells in an antigen-dependent fashion in ^51^Cr release assays and secreted immunostimulatory cytokines upon encounter of TEM8 positive cells. There was no reactivity against TEM8 negative cell lines. No cytotoxicity or cytokine release was exhibited by T cells expressing an irrelevant (CD19 specific) CAR or non-transduced T cells from the same blood donor. We are currently testing this strategy in a vascularized orthotopic breast cancer murine model.

## Conclusion

TEM8 specific CAR T cells could serve as a tumor and vascular-targeted immunotherapeutic modality for triple-negative breast cancer.
